# Supplementary material for: Discovery and characterization of a Gram-positive Pel polysaccharide biosynthetic gene cluster
Source: PLoS Pathog. 2020 Apr 1;16(4):e1008281. doi: 10.1371/journal.ppat.1008281 (PMC7112168; doi:10.1371/journal.ppat.1008281)
Supplement: S1 Fig — PelF is a glycosyltransferase that polymerizes UDP-GalNAc and UDP-GlcNAc to generate the Pel polysaccharide (light pink triangles are acetyl groups, red hexagons are GalN/GlcN monosaccharide units, dark red teardrops are UDP). Once synthesized, Pel is transported across the inner membrane via PelD, PelE, and/or PelG. In the periplasm, PelA interacts with PelB to partially deacetylate the polymer and generate mature Pel, which is subsequently exported across the outer membrane through the β-barrel porin domain of PelB. PelC forms a dodecameric funnel that helps to channel Pel towards the PelB pore. Pel biosynthesis requires binding of the nucleotide second messenger c-di-GMP to the inhibitory site of the cytoplasmic GGDEF domain of PelD. C, cytoplasm; IM, inner membrane; P, periplasmic space; PG, peptidoglycan; OM, outer membrane; c-di-GMP, cyclic-3’,5’-dimeric guanosine monophosphate. (PDF) [file ppat.1008281.s001.pdf]

Figure S1

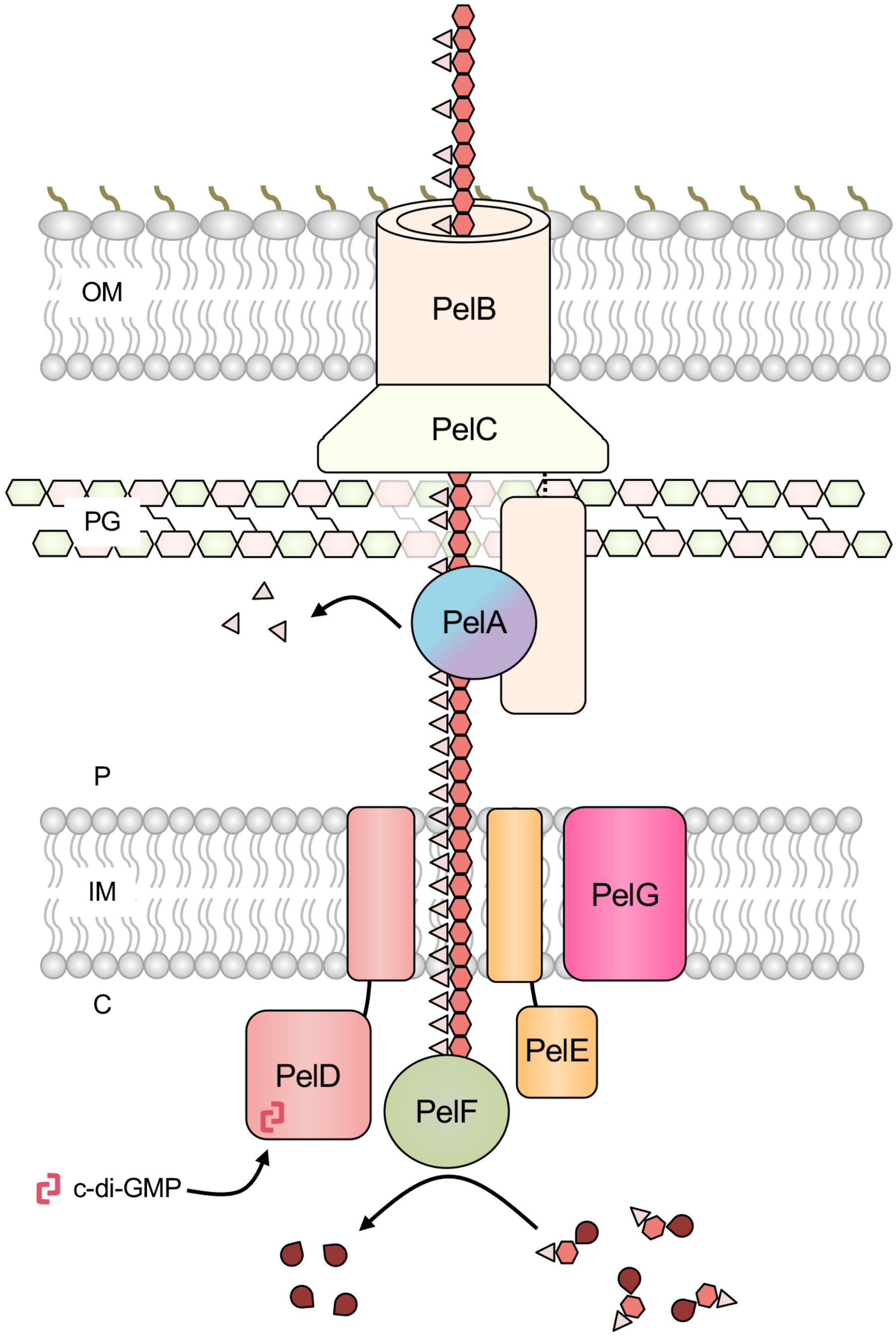

<u>Figure S1:</u> Model of *P. aeruginosa* Pel biosynthesis. PelF is a glycosyltransferase that polymerizes UDP-GalNAc and UDP-GlcNAc to generate the Pel polysaccharide (light pink triangles are acetyl groups, red hexagons are GalN/GlcN monosaccharide units, dark red teardrops are UDP). Once synthesized, Pel is transported across the inner membrane via PelD, PelE, and/or PelG. In the periplasm, PelA interacts with the soluble tetratricopeptide repeat (TPR) domain of PelB to partially deacetylate the polymer and generate mature Pel, which is subsequently exported across the outer membrane through the β-barrel porin domain of PelB. PelC forms a dodecameric funnel that helps to channel Pel towards the PelB pore. Pel biosynthesis requires binding of the nucleotide second messenger c-di-GMP to the inhibitory site of the cytoplasmic GGDEF domain of PelD. C, cytoplasm; IM, inner membrane; P, periplasmic space; PG, peptidoglycan; OM, outer membrane; c-di-GMP, cyclic-3',5'-dimeric guanosine monophosphate.
