# Supplementary material for: Discovery and characterization of a Gram-positive Pel polysaccharide biosynthetic gene cluster
Source: PLoS Pathog. 2020 Apr 1;16(4):e1008281. doi: 10.1371/journal.ppat.1008281 (PMC7112168; doi:10.1371/journal.ppat.1008281)
Supplement: S2 Fig — Predicted domains are depicted as rectangles. Individual domains and overall domain architectures are drawn to scale. The length of each protein is indicated to the right of the respective diagram. Rectangle colours correspond to predicted domain functions, which are listed in the legend at the bottom. Residues from SDR fold that are required for co-factor binding (left) and epimerase activity (right) are shown within the orange rectangle, and are highlighted in bold if they match the consensus sequence for the prescribed function. Residues from the GGDEF fold that are required for c-di-GMP binding to the inhibitory site (left) and diguanylate cyclase activity (righ) are shown within the red rectangle, and are highlighted in bold if they match the consensus sequence required for the prescribed function. TM, transmembrane; GAF, cGMP-specific phosphodiesterase, adenylyl cyclase and FhlA domain; GGDEF, diguanylate cyclase-like domain; SDR, short chain dehydrogenase/reductase. (PDF) [file ppat.1008281.s002.pdf]

Figure S2

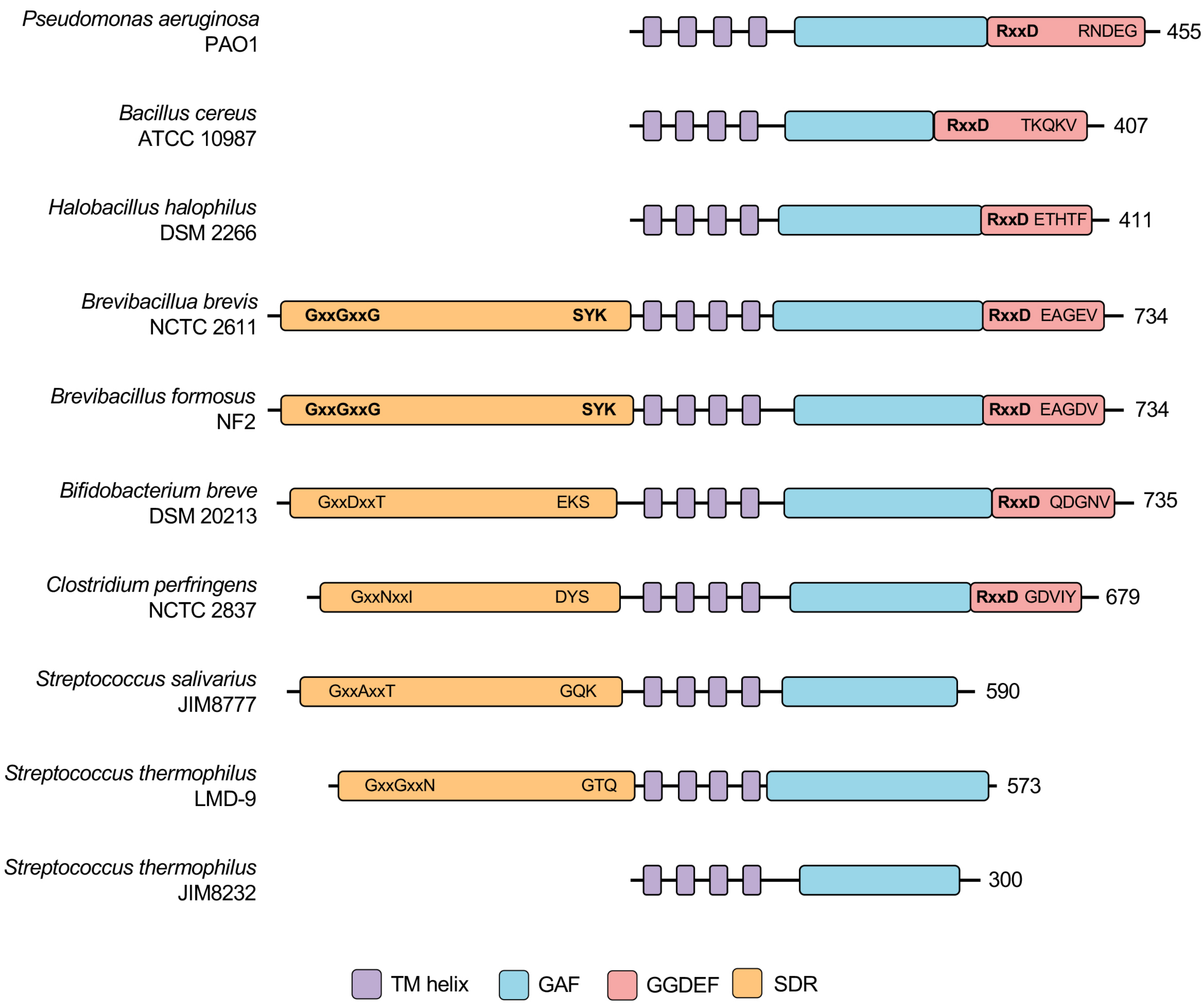

<u>Figure S2:</u> Predicted domain architectures of representative PelD proteins from the indicated organisms. Predicted domains are depicted as rectangles. Individual domains and overall domain architectures are drawn to scale. The length of each protein is indicated to the right of the respective diagram. Rectangle colours correspond to predicted domain functions, which are listed in the legend at the bottom. Residues from the SDR fold that are required for co-factor binding (left side of orange rectangle) and epimerase activity (right side of orange rectangle) are shown, and are highlighted in bold if they match the sequence required for the prescribed function. Residues from the GGDEF fold that are required for c-di-GMP binding to the inhibitory site (left side of red rectangle) and diguanylate cyclase activity (right side of red rectangle) are shown, and are highlighted in bold if they match the sequence required for the prescribed function. TM, transmembrane; GAF, cGMP-specific phosphodiesterase, adenylyl cyclase and FhlA domain; GGDEF, diguanylate cyclase-like domain; SDR, short chain dehydrogenase/reductase.
