# Supplementary material for: Discovery and characterization of a Gram-positive Pel polysaccharide biosynthetic gene cluster
Source: PLoS Pathog. 2020 Apr 1;16(4):e1008281. doi: 10.1371/journal.ppat.1008281 (PMC7112168; doi:10.1371/journal.ppat.1008281)
Supplement: S3 Fig — Operons with a conserved accessory gene architecture identified in Bacilli are shown at the top, while operons with divergent architectures are shown at the bottom. Open reading frames are represented as arrows, with the directionality of transcription indicated by the arrow direction. Open reading frames and overall operon architectures are drawn to scale. Arrow colours correspond to predicted protein functions, which are listed in the legend at the bottom. PelAH, PelA hydrolase-like domain; PelADA, PelA deacetylase-like domain. (PDF) [file ppat.1008281.s003.pdf]

Figure S3

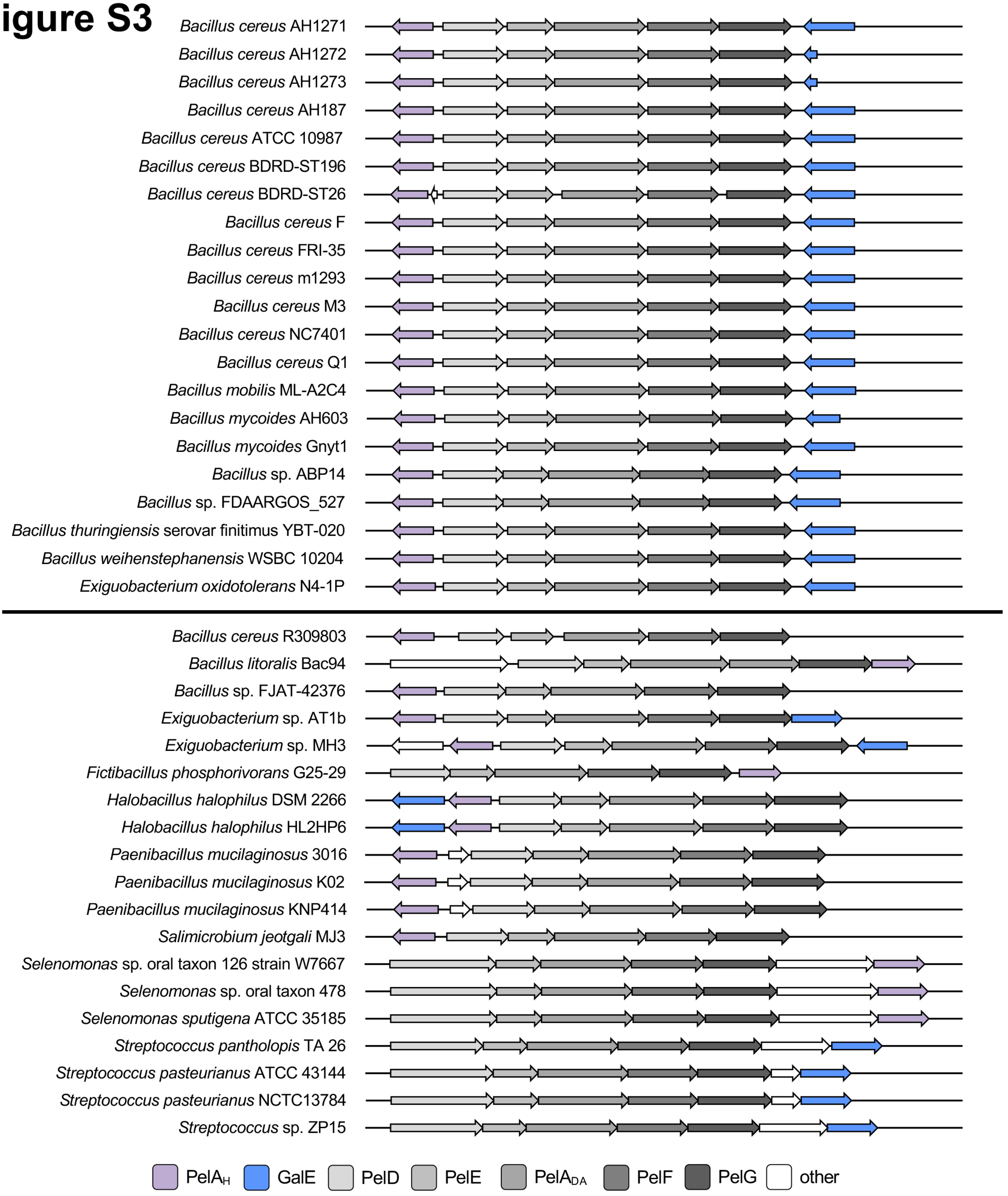

<u>Figure S3:</u> Representative operon architectures containing the accessory genes *galE* and *pelA$_H$*. Operons with a conserved accessory gene architecture identified in Bacilli are shown at the top, while operons with divergent architectures are shown at the bottom. Open reading frames are represented as arrows, with the directionality of transcription indicated by the arrow direction. Open reading frames and overall operon architectures are drawn to scale. Arrow colours correspond to predicted protein functions, which are listed in the legend at the bottom. PelA$_H$, PelA hydrolase-like domain; PelA$_{DA}$, PelA deacetylase-like domain.
