# Supplementary material for: Discovery and characterization of a Gram-positive Pel polysaccharide biosynthetic gene cluster
Source: PLoS Pathog. 2020 Apr 1;16(4):e1008281. doi: 10.1371/journal.ppat.1008281 (PMC7112168; doi:10.1371/journal.ppat.1008281)
Supplement: S4 Fig — Operons with a conserved accessory gene architecture are shown at the top, while operons with divergent architectures are shown at the bottom. Open reading frames are represented as arrows, with the directionality of transcription indicated by the arrow direction. Open reading frames and overall operon architectures are drawn to scale. Arrow colours correspond to predicted protein functions, which are listed in the legend at the bottom. PelADA, PelA deacetylase-like domain; PPP, poly-phosphate polymerase; DUF, domain of unknown function. (PDF) [file ppat.1008281.s004.pdf]

Figure S4

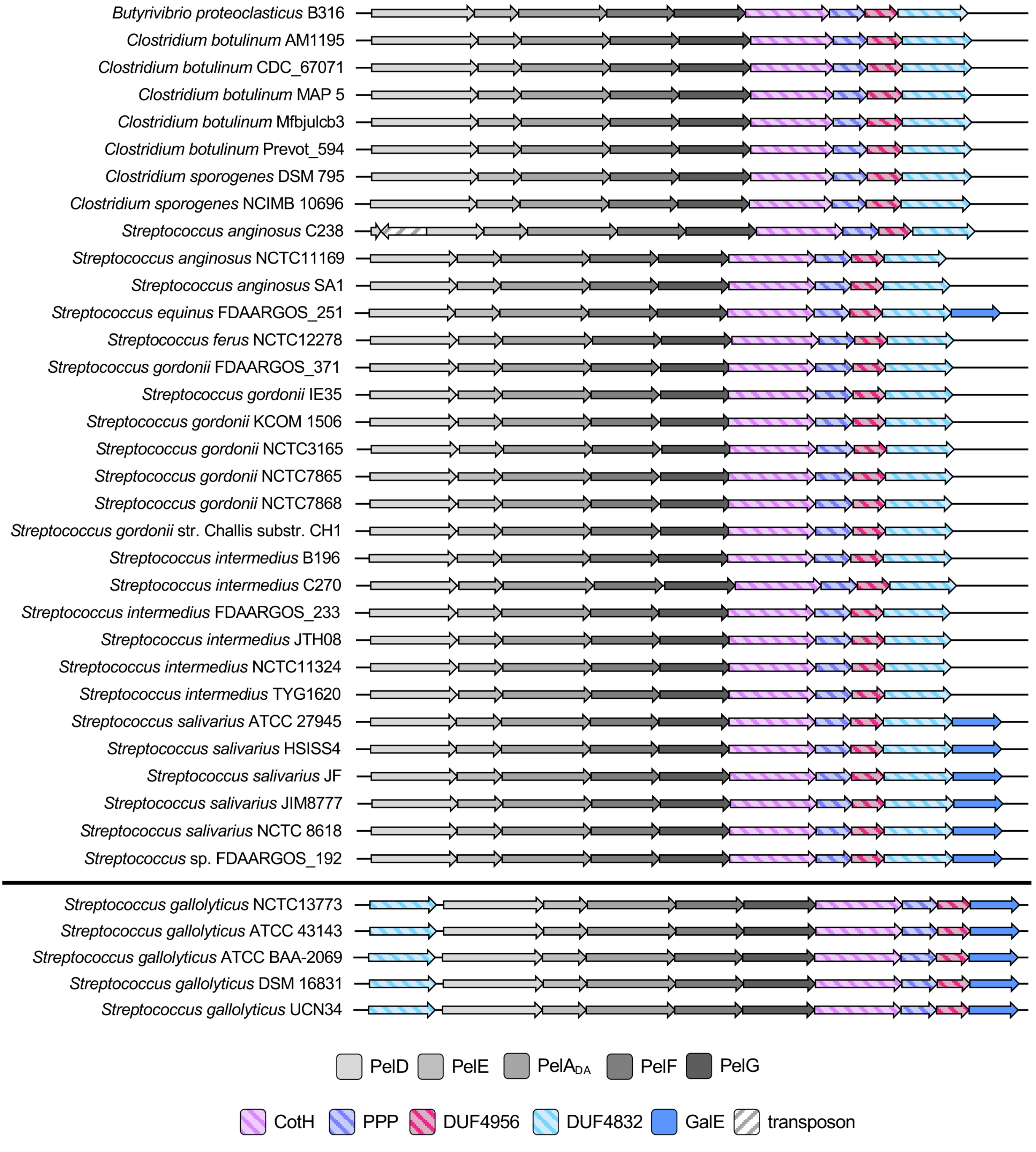

<u>**Figure S4:**</u> **Representative operon architectures identified in Streptococci and Clostridia containing the accessory genes *cotH*, *PPP*, *DUF4956*, and *DUF4832*.** Operons with a conserved accessory gene architecture are shown at the top, while operons with divergent architectures are shown at the bottom. Open reading frames are represented as arrows, with the directionality of transcription indicated by the arrow direction. Open reading frames and overall operon architectures are drawn to scale. Arrow colours correspond to predicted protein functions, which are listed in the legend at the bottom. PelA$_{DA}$, PelA deacetylase-like domain; PPP, polyphosphate polymerase; DUF, domain of unknown function.
