# Supplementary material for: Discovery and characterization of a Gram-positive Pel polysaccharide biosynthetic gene cluster
Source: PLoS Pathog. 2020 Apr 1;16(4):e1008281. doi: 10.1371/journal.ppat.1008281 (PMC7112168; doi:10.1371/journal.ppat.1008281)
Supplement: S5 Fig — Open reading frames are represented as arrows, with the directionality of transcription indicated by the arrow direction. Open reading frames and overall operon architectures are drawn to scale. Arrow colours correspond to predicted protein functions, which are listed in the legend at the bottom. PelADA, PelA deacetylase-like domain; PPP, poly-phosphate polymerase; DUF, domain of unknown function; PDE-DGC, phosphodiesterase/diguanylate cyclase-like domain. (PDF) [file ppat.1008281.s005.pdf]

Figure S5

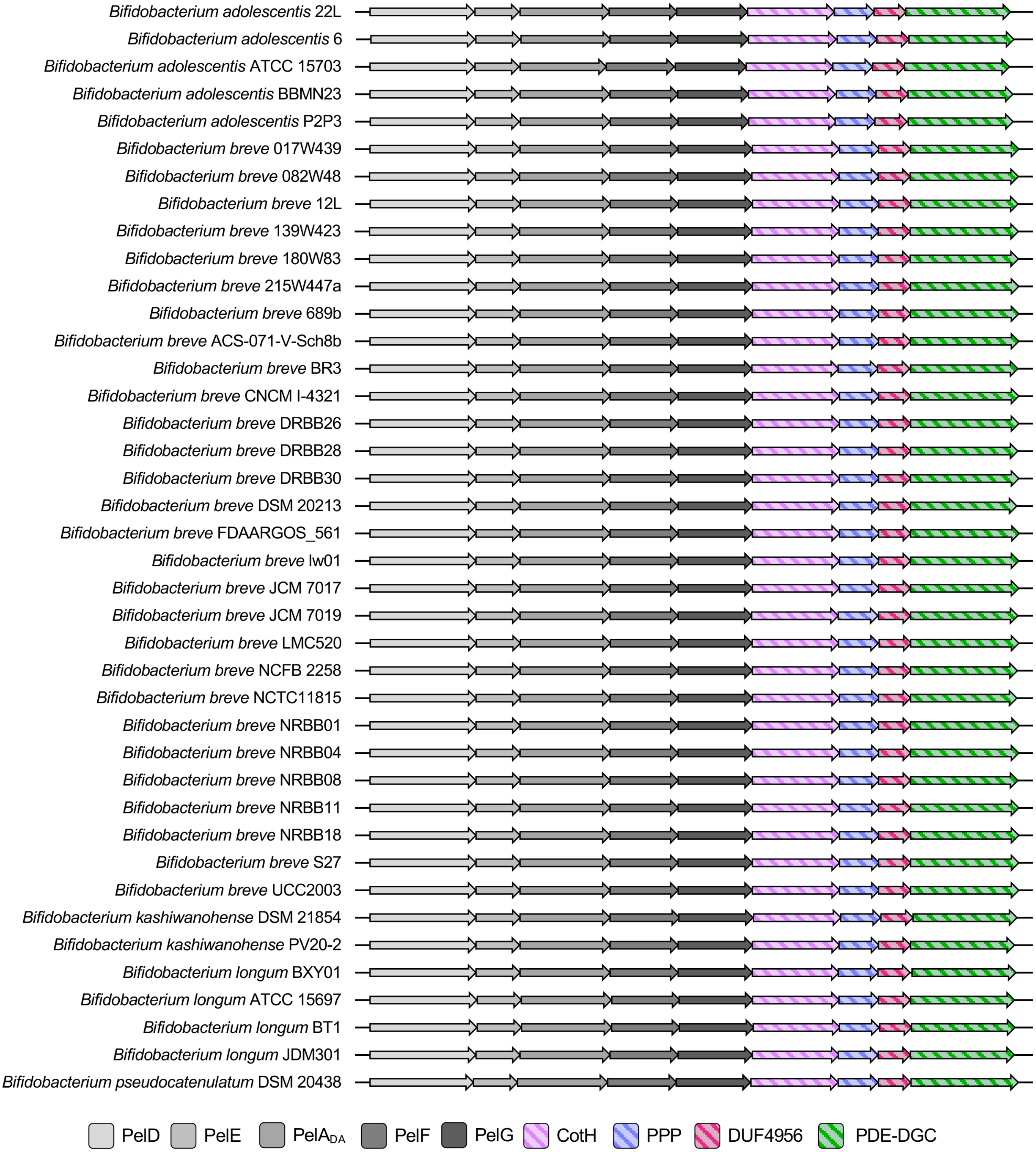

<u>Figure S5:</u> Representative operons with a conserved architecture identified in Bifidobacteria containing the accessory genes *cotH*, *PPP, DUF4956*, and *DGC/PDE*. Open reading frames are represented as arrows, with the directionality of transcription indicated by the arrow direction. Open reading frames and overall operon architectures are drawn to scale. Arrow colours correspond to predicted protein functions, which are listed in the legend at the bottom. PelA$_{DA}$, PelA deacetylase-like domain; PPP, poly-phosphate polymerase; DUF, domain of unknown function; PDE-DGC, phosphodiesterase/diguanylate cyclase-like domain.
