# Supplementary material for: Discovery and characterization of a Gram-positive Pel polysaccharide biosynthetic gene cluster
Source: PLoS Pathog. 2020 Apr 1;16(4):e1008281. doi: 10.1371/journal.ppat.1008281 (PMC7112168; doi:10.1371/journal.ppat.1008281)
Supplement: S7 Fig — Open reading frames are represented as arrows, with the directionality of transcription indicated by the arrow direction. Open reading frames and overall operon architectures are drawn to scale. The predicted functions of the protein products of each gene are indicated above or below that gene. GGDEF, diguanylate cyclase-like domain; DUF, domain of unknown function; GT1, glycosyltransferase family 1; GT2, glycosyltransferase family 2; PTase, phosphotransferase; NTase, nucleotidyltransferase. (PDF) [file ppat.1008281.s007.pdf]

Figure S7

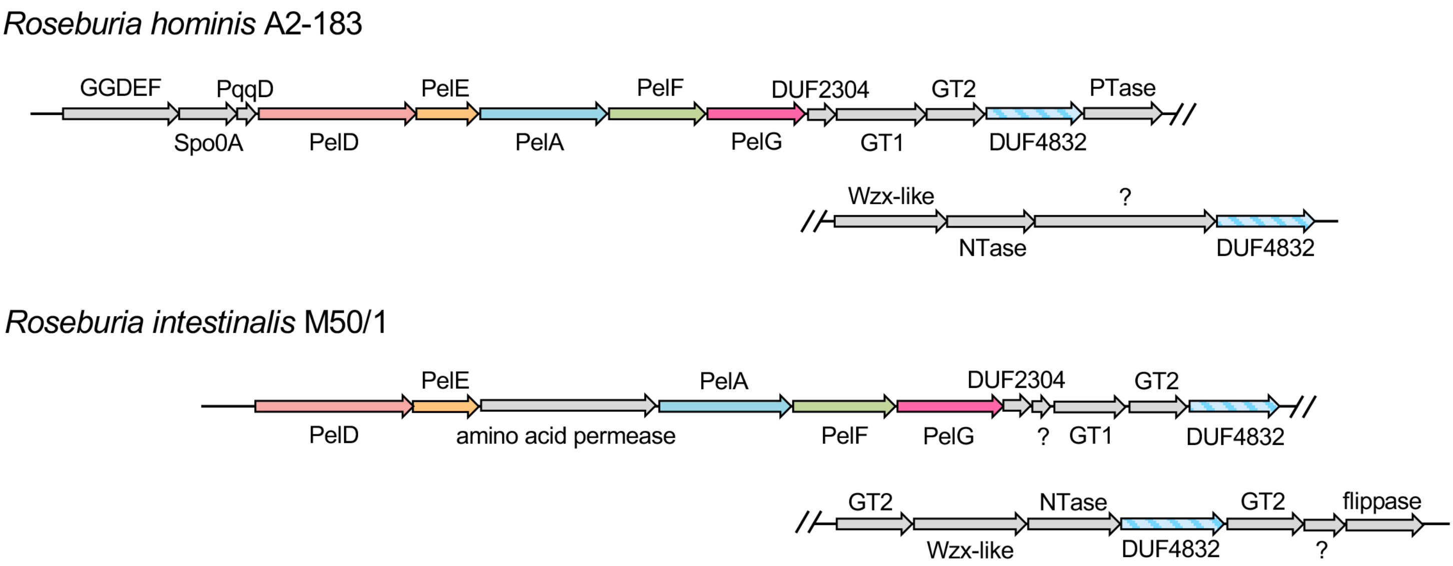

<u>**Figure S7:**</u> **Operon architectures from *Roseburia* species, which have the putative *pel* genes within the context of a larger operon likely involved in capsule or teichoic acid biosynthesis.** Open reading frames are represented as arrows, with the directionality of transcription indicated by the arrow direction. Open reading frames and overall operon architectures are drawn to scale. The predicted functions of the protein products of each gene are indicated above or below that gene. GGDEF, diguanylate cyclase-like domain; DUF, domain of unknown function; GT1, glycosyltransferase family 1; GT2, glycosyltransferase family 2; PTase, phosphotransferase; NTase, nucleotidyltransferase.
