# Supplementary material for: Discovery and characterization of a Gram-positive Pel polysaccharide biosynthetic gene cluster
Source: PLoS Pathog. 2020 Apr 1;16(4):e1008281. doi: 10.1371/journal.ppat.1008281 (PMC7112168; doi:10.1371/journal.ppat.1008281)
Supplement: S8 Fig — (top) Biofilm formation by the indicated strains of B. cereus ATCC 10987 assessed by the crystal violet assay. Error bars represent the standard error of the mean of six independent trials. Statistical significance was determined using one-way analysis of variance with Dunn’s multiple comparison. ****, p < 0.0001. VC, empty vector control. (middle) Air-liquid interface (pellicle) biofilm formation by the indicated strains of B. cereus ATCC 10987 in borosilicate glass tubes. The pellicle is indicated by the black arrow. (bottom) Staining of biomass adherent to the walls of the borosilicate glass tubes pictured above with crystal violet. Non-adherent cells and media were washed from the glass tube before crystal violet staining. (PDF) [file ppat.1008281.s008.pdf]

Figure S8

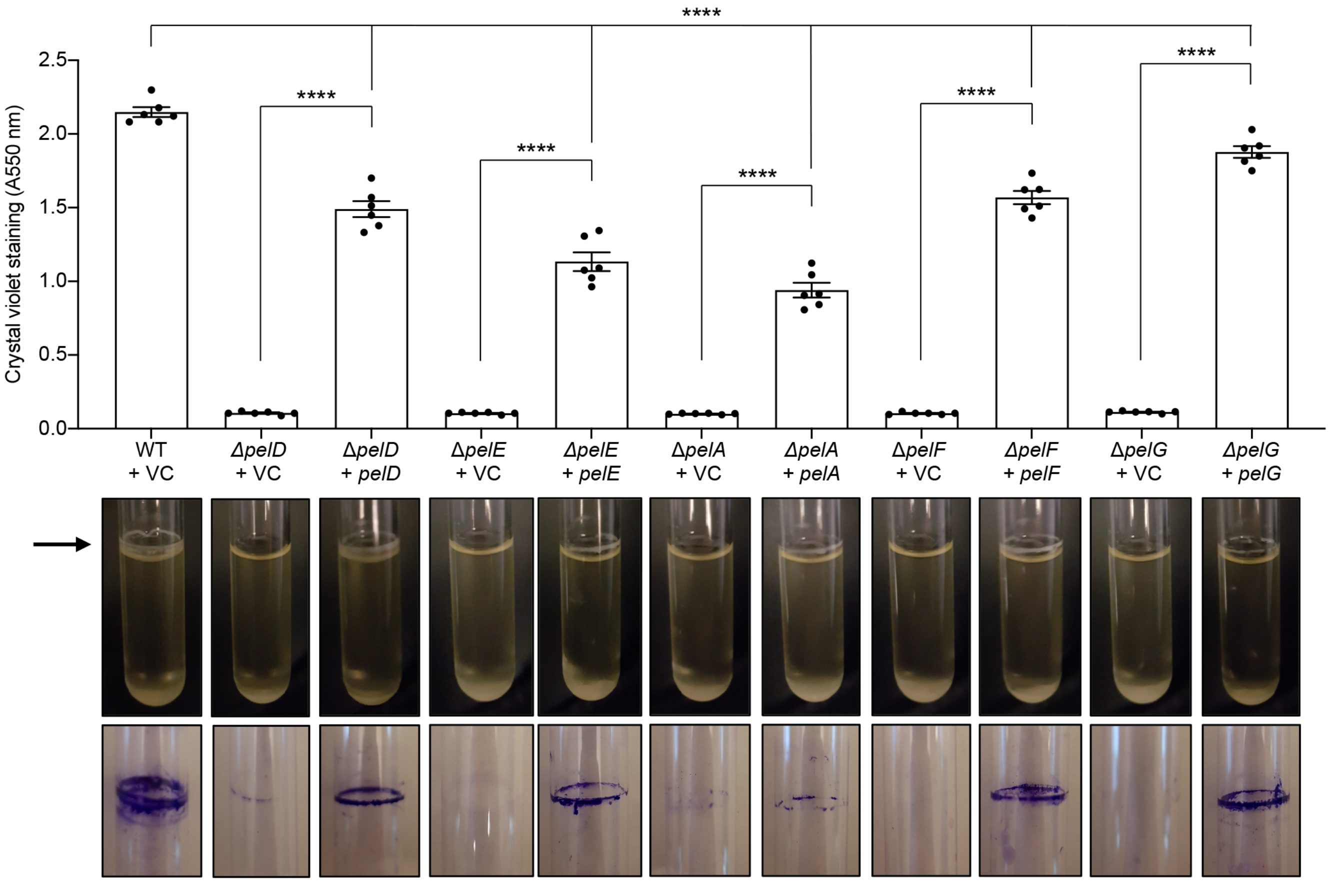

<u>Figure S8:</u> Complementation of *$pelDEA_{DA}FG$* deletion mutants restores biofilm formation. (*top*) Biofilm formation by the indicated strains of *B. cereus* ATCC 10987 assessed by the crystal violet assay. Error bars represent the standard error of the mean of six independent trials. Statistical significance was determined using one-way analysis of variance with Dunn's multiple comparison. ****, $p < 0.0001$. VC, empty vector control. (*middle*) Air-liquid interface (pellicle) biofilm formation by the indicated strains of *B. cereus* ATCC 10987 in borosilicate glass tubes. The pellicle is indicated by the black arrow. (*bottom*) Staining of biomass adherent to the walls of the borosilicate glass tubes pictured above with crystal violet. Non-adherent cells and media were washed from the glass tube before crystal violet staining.
