# Supplementary material for: Discovery and characterization of a Gram-positive Pel polysaccharide biosynthetic gene cluster
Source: PLoS Pathog. 2020 Apr 1;16(4):e1008281. doi: 10.1371/journal.ppat.1008281 (PMC7112168; doi:10.1371/journal.ppat.1008281)
Supplement: S9 Fig — (A-B) Dose-response curves generated by the exogenous application of the indicated glycoside hydrolase enzyme to pre-formed biofilms of the indicated strains. (A) Application of exogenous PelAHBc to pre-formed P. aeruginosa PAO1 biofilms. (B) Application of exogenous PgaBBb to pre-formed B. cereus ATCC 10987 biofilms. Error bars represent the standard error of the mean of six independent trials. (PDF) [file ppat.1008281.s009.pdf]

Figure S9

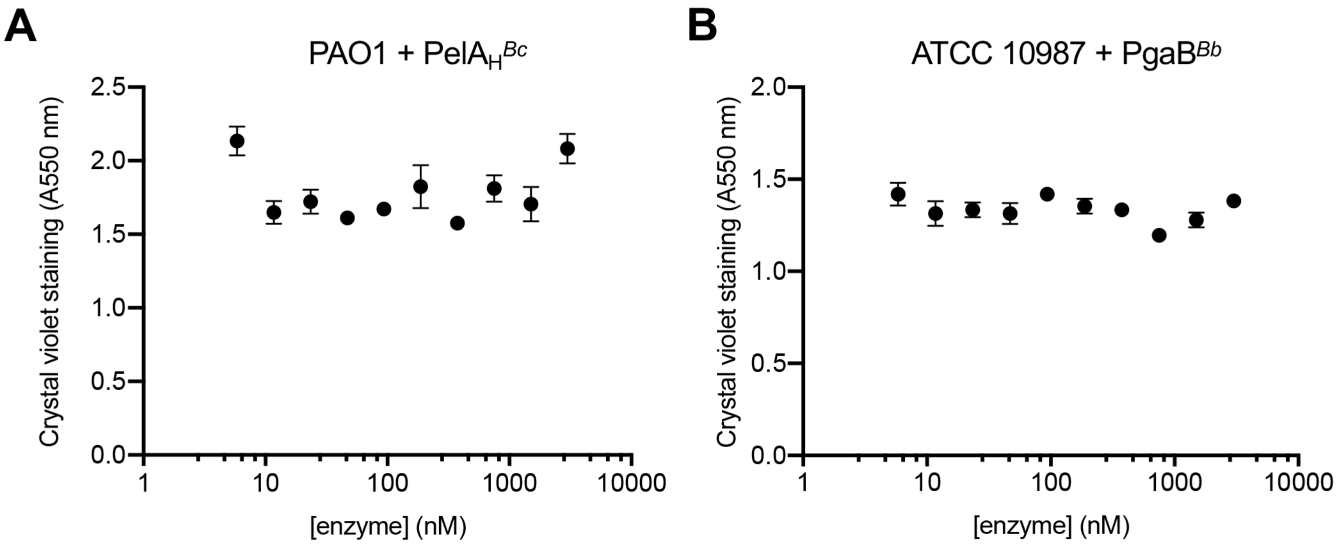

<u>Figure S9:</u> PelA$_H^{Bc}$ specifically disrupts Pel-dependent biofilms. (A-B) Dose-response curves generated by the exogenous application of the indicated glycoside hydrolase enzyme to pre-formed biofilms of the indicated strains. (A) Application of exogenous PelA$_H^{Bc}$ to pre-formed *P. aeruginosa* PAO1 biofilms. (B) Application of exogenous PgaB^{Bb} to pre-formed *B. cereus* ATCC 10987 biofilms. Error bars represent the standard error of the mean of six independent trials.
