# Supplementary material for: Discovery and characterization of a Gram-positive Pel polysaccharide biosynthetic gene cluster
Source: PLoS Pathog. 2020 Apr 1;16(4):e1008281. doi: 10.1371/journal.ppat.1008281 (PMC7112168; doi:10.1371/journal.ppat.1008281)
Supplement: S1 Table — List of species, and PelG and Genome accession numbers. (DOCX) [file ppat.1008281.s010.docx]

**Table S1: Complete list of Gram-positive organisms with *pel*-like operons.**

| **Species*** | **PelG Protein Accession** | **Genome Accession** |
| --- | --- | --- |
| *Bacillus cereus* AH1271 | WP_000907287.1 | NZ_CM000739.1 |
| *Bacillus cereus* AH1272 | WP_000907285.1 | NZ_CM000740.1 |
| *Bacillus cereus* AH1273 | WP_000907285.1 | NZ_CM000741.1 |
| *Bacillus cereus* AH187 | WP_000907295.1 | NC_011658.1 |
| ***Bacillus cereus* ATCC 10987** | **WP_000907294.1** | **NC_003909.8** |
| *Bacillus cereus* BDRD-ST196 | WP_033709612.1 | NZ_CM000725.1 |
| *Bacillus cereus* BDRD-ST26 | EEK97794.1 | CM000724.1 |
| *Bacillus cereus* F | WP_016719067.1 | NZ_CM001787.1 |
| *Bacillus cereus* FRI-35 | WP_014893388.1 | NC_018491.1 |
| *Bacillus cereus* m1293 | WP_000907291.1 | NZ_CM000714.1 |
| *Bacillus cereus* M3 | WP_016719067.1 | NZ_CP016316.1 |
| *Bacillus cereus* NC7401 | WP_000907295.1 | NC_016771.1 |
| *Bacillus cereus* Q1 | WP_000907290.1 | NC_011969.1 |
| *Bacillus cereus* R309803 | EEK76039.1 | CM000720.1 |
| ***Bacillus litoralis* Bac94** | **WP_121663553.1** | **NZ_CP033043.1** |
| ***Bacillus mobilis* ML-A2C4** | **WP_000907287.1** | **NZ_CP031443.1** |
| ***Bacillus mycoides* AH603** | **WP_033707410.1** | **NZ_CM000737.1** |
| *Bacillus mycoides* Gnyt1 | WP_002167287.1 | NZ_CP020743.1 |
| ***Bacillus* sp. ABP14** | **WP_070807265.1** | **NZ_CP017016.1** |
| ***Bacillus* sp. FDAARGOS_527** | **WP_063246657.1** | **NZ_CP033795.1** |
| ***Bacillus* sp. FJAT-42376** | **WP_123918462.1** | **NZ_CP033906.1** |
| ***Bacillus thuringiensis* serovar finitimus YBT-020** | **WP_000907289.1** | **NC_017200.1** |
| ***Bacillus weihenstephanensis* WSBC 10204** | **WP_033709612.1** | **NZ_CP009746.1** |
| *Bifidobacterium adolescentis* 22L | WP_038444142.1 | NZ_CP007443.1 |
| *Bifidobacterium adolescentis* 6 | WP_039774842.1 | NZ_CP023005.1 |
| ***Bifidobacterium adolescentis* ATCC 15703** | **WP_011742867.1** | **NC_008618.1** |
| *Bifidobacterium adolescentis* BBMN23 | WP_039774842.1 | NZ_CP010437.1 |
| *Bifidobacterium adolescentis* P2P3 | WP_039774842.1 | NZ_CP024959.1 |
| *Bifidobacterium breve* 017W439 | WP_052822180.1 | NZ_CP021554.1 |
| *Bifidobacterium breve* 082W48 | WP_106622134.1 | NZ_CP021555.1 |
| *Bifidobacterium breve* 12L | WP_019727643.1 | NZ_CP006711.1 |
| *Bifidobacterium breve* 139W423 | WP_021648898.1 | NZ_CP021556.1 |
| *Bifidobacterium breve* 180W83 | WP_106630814.1 | NZ_CP021557.1 |
| *Bifidobacterium breve* 215W447a | WP_106641225.1 | NZ_CP021558.1 |
| *Bifidobacterium breve* 689b | WP_025331928.1 | NZ_CP006715.1 |
| *Bifidobacterium breve* ACS-071-V-Sch8b | WP_014483227.1 | NC_017218.1 |
| *Bifidobacterium breve* BR3 | WP_052789711.1 | NZ_CP010413.1 |
| *Bifidobacterium breve* CNCM I-4321 | WP_106642177.1 | NZ_CP021559.1 |
| *Bifidobacterium breve* DRBB26 | WP_003833139.1 | NZ_CP021390.1 |
| *Bifidobacterium breve* DRBB27 | WP_106621239.1 | NZ_CP021552.1 |
| *Bifidobacterium breve* DRBB28 | WP_003833139.1 | NZ_CP021553.1 |
| *Bifidobacterium breve* DRBB29 | WP_106621239.1 | NZ_CP023198.1 |
| *Bifidobacterium breve* DRBB30 | WP_106642177.1 | NZ_CP023199.1 |
| ***Bifidobacterium breve* DSM 20213 = JCM 1192** | **WP_003827899.1** | **NZ_AP012324.1** |
| *Bifidobacterium breve* FDAARGOS_561 | WP_003827899.1 | NZ_CP033841.1 |
| *Bifidobacterium breve* lw01 | WP_016462023.1 | NZ_CP034192.1 |
| *Bifidobacterium breve* JCM 7017 | WP_025300916.1 | NZ_CP006712.1 |
| *Bifidobacterium breve* JCM 7019 | WP_025220954.1 | NZ_CP006713.1 |
| *Bifidobacterium breve* LMC520 | WP_077149301.1 | NZ_CP019596.1 |
| *Bifidobacterium breve* NCFB 2258 | WP_015438200.1 | NZ_CP006714.1 |
| *Bifidobacterium breve* NCTC 11815 | WP_003827899.1 | NZ_LR134348.1 |
| *Bifidobacterium breve* NRBB01 | WP_003827899.1 | NZ_CP021384.1 |
| *Bifidobacterium breve* NRBB02 | WP_106622507.1 | NZ_CP021385.1 |
| *Bifidobacterium breve* NRBB04 | WP_015438200.1 | NZ_CP021386.1 |
| *Bifidobacterium breve* NRBB08 | WP_106622507.1 | NZ_CP023192.1 |
| *Bifidobacterium breve* NRBB09 | WP_106629152.1 | NZ_CP021387.1 |
| *Bifidobacterium breve* NRBB11 | WP_106628600.1 | NZ_CP021388.1 |
| *Bifidobacterium breve* NRBB18 | WP_106622507.1 | NZ_CP023193.1 |
| *Bifidobacterium breve* NRBB19 | WP_106622507.1 | NZ_CP023194.1 |
| *Bifidobacterium breve* NRBB20 | WP_106622507.1 | NZ_CP023195.1 |
| *Bifidobacterium breve* NRBB27 | WP_106622507.1 | NZ_CP023196.1 |
| *Bifidobacterium breve* NRBB49 | WP_106622507.1 | NZ_CP023197.1 |
| *Bifidobacterium breve* NRBB50 | WP_106628600.1 | NZ_CP021391.1 |
| *Bifidobacterium breve* NRBB51 | WP_019727643.1 | NZ_CP021392.1 |
| *Bifidobacterium breve* NRBB52 | WP_080867748.1 | NZ_CP021393.1 |
| *Bifidobacterium breve* NRBB56 | WP_016462023.1 | NZ_CP021394.1 |
| *Bifidobacterium breve* NRBB57 | WP_052822180.1 | NZ_CP021389.1 |
| *Bifidobacterium breve* S27 | WP_025341481.1 | NZ_CP006716.1 |
| *Bifidobacterium breve* UCC2003 | WP_015438200.1 | NC_020517.1 |
| ***Bifidobacterium kashiwanohense* JCM 15439 = DSM 21854** | **WP_033500884.1** | **NZ_AP012327.1** |
| *Bifidobacterium kashiwanohense* PV20-2 | WP_039197257.1 | NZ_CP007456.1 |
| *Bifidobacterium longum* BXY01 | WP_013141166.1 | NZ_CP008885.1 |
| ***Bifidobacterium longum* subsp. infantis ATCC 15697 = JCM 1222 = DSM 20088** | **WP_014484651.1** | **NC_017219.1** |
| *Bifidobacterium longum* subsp. infantis BT1 | WP_060620711.1 | NZ_CP010411.1 |
| *Bifidobacterium longum* subsp. longum JDM301 | WP_013141166.1 | NC_014169.1 |
| ***Bifidobacterium pseudocatenulatum* DSM 20438 = JCM 1200 = LMG 10505** | **WP_004219882.1** | **NZ_AP012330.1** |
| *Brevibacillus brevis* DZQ7 | WP_048031022.1 | NZ_CP030117.1 |
| *Brevibacillus brevis* NBRC 100599 | WP_012684303.1 | NC_012491.1 |
| ***Brevibacillus brevis* NCTC 2611** | **WP_106652439.1** | **NZ_LR134338.1** |
| *Brevibacillus brevis* X23 | WP_017249674.1 | NZ_CP023474.1 |
| ***Brevibacillus formosus* NF2** | **WP_088910547.1** | **NZ_CP018145.1** |
| ***Butyrivibrio fibrisolvens* INBov1** | **WP_110073161.1** | **NZ_CM009896.1** |
| ***Butyrivibrio proteoclasticus* B316** | **WP_013281180.1** | **NC_014387.1** |
| *Clostridium botulinum* AM1195 | WP_061328351.1 | NZ_CP013701.1 |
| *Clostridium botulinum* CDC_67071 | WP_096043970.1 | NZ_CP013242.1 |
| *Clostridium botulinum* MAP 5 | WP_106899782.1 | NZ_CP027781.1 |
| *Clostridium botulinum* Mfbjulcb8 | WP_061328351.1 | NZ_CP027780.1 |
| ***Clostridium botulinum* Prevot_594** | **WP_040108159.1** | **NZ_CP006902.1** |
| *Clostridium perfringens* F262 | WP_003481250.1 | NZ_CM001477.1 |
| *Clostridium perfringens* FORC_003 | WP_003466749.1 | NZ_CP009557.1 |
| *Clostridium perfringens* FORC_025 | WP_070956832.1 | NZ_CP013101.1 |
| *Clostridium perfringens* JP838 | WP_061427915.1 | NZ_CP010994.1 |
| *Clostridium perfringens* JXJA17 | WP_003452556.1 | NZ_CP028149.1 |
| ***Clostridium perfringens* NCTC 2837** | **WP_003473144.1** | **NZ_LS483461.1** |
| ***Clostridium sporogenes* DSM 795** | **WP_003491425.1** | **NZ_CP011663.1** |
| *Clostridium sporogenes* NCIMB 10696 | WP_003491425.1 | NZ_CP009225.1 |
| ***Collinsella aerofaciens* C11** | **WP_117798150.1** | **NZ_CP024960.1** |
| *Collinsella aerofaciens* indica | WP_099431625.1 | NZ_CP024160.1 |
| ***Exiguobacterium oxidotolerans* N4-1P** | **WP_088837913.1** | **NZ_CP022236.1** |
| ***Exiguobacterium* sp. AT1b** | **WP_012727256.1** | **NC_012673.1** |
| ***Exiguobacterium* sp. MH3** | **WP_023469423.1** | **NC_022794.1** |
| ***Fictibacillus phosphorivorans* G25-29** | **WP_066398125.1** | **NZ_CP015378.1** |
| ***Halobacillus halophilus* DSM 2266** | **WP_014644340.1** | **NC_017668.1** |
| *Halobacillus halophilus* HL2HP6 | WP_014644340.1 | NZ_CP022106.1 |
| *Paenibacillus mucilaginosus* 3016 | WP_013920028.1 | NC_016935.1 |
| *Paenibacillus mucilaginosus* K02 | WP_013920028.1 | NC_017672.3 |
| ***Paenibacillus mucilaginosus* KNP414** | **WP_013920028.1** | **NC_015690.1** |
| ***Paenibacillu*s sp. BD3526** | **WP_060531382.1** | **NZ_CP013023.1** |
| ***Romboutsia* sp. Frifi** | **CEI72691.1** | **LN650648.1** |
| ***Roseburia hominis* A2-183** | **WP_014079902.1** | **NC_015977.1** |
| ***Roseburia intestinalis* M50/1** | **CBL09539.1** | **FP929049.1** |
| ***Rubrobacter xylanophilus* DSM 9941** | **WP_011565595.1** | **NC_008148.1** |
| ***Salimicrobium jeotgali* MJ3** | **WP_008592356.1** | **NZ_CP011361.2** |
| ***Selenomonas* sp. oral taxon 126 strain W7667** | **WP_066844117.1** | **NZ_CP016201.1** |
| ***Selenomona*s sp. oral taxon 478** | **WP_050342638.1** | **NZ_CP012071.1** |
| ***Selenomonas sputigena* ATCC 35185** | **WP_006193285.1** | **NC_015437.1** |
| *Streptococcus anginosus* C238 | WP_003035167.1 | NC_022239.1 |
| ***Streptococcus anginosus* NCTC 11169** | **WP_126407817.1** | **NZ_LR134288.1** |
| *Streptococcus anginosus* SA1 | WP_004224623.1 | NZ_CP007573.1 |
| ***Streptococcus equinus* FDAARGOS_251** | **WP_107373002.1** | **NZ_CP020438.1** |
| ***Streptococcus ferus* NCTC 12278** | **WP_018030127.1** | **NZ_LS483343.1** |
| *Streptococcus gallolyticus* NCTC 13773 | WP_077496569.1 | NZ_LS483409.1 |
| *Streptococcus gallolyticus* subsp. gallolyticus ATCC 43143 | WP_012961717.1 | NC_017576.1 |
| *Streptococcus gallolyticus* subsp. gallolyticus ATCC BAA-2069 | WP_013642905.1 | NC_015215.1 |
| ***Streptococcus gallolyticus* subsp. gallolyticus DSM 16831** | **WP_077496569.1** | **NZ_CP018822.1** |
| *Streptococcus gallolyticus* UCN34 | WP_012961717.1 | NC_013798.1 |
| *Streptococcus gordonii* FDAARGOS_371 | WP_061595675.1 | NZ_CP023511.1 |
| *Streptococcus gordonii* IE35 | WP_046165000.1 | NZ_CP017295.1 |
| *Streptococcus gordonii* KCOM 1506 (= ChDC B679) | WP_053794170.1 | NZ_CP012648.1 |
| *Streptococcus gordonii* NCTC 3165 | WP_111723252.1 | NZ_LS483375.1 |
| ***Streptococcus gordonii* NCTC 7865** | **WP_060553094.1** | **NZ_LS483341.1** |
| *Streptococcus gordonii* NCTC 7868 | WP_011999856.1 | NZ_LR134291.1 |
| *Streptococcus gordonii* str. Challis substr. CH1 | WP_011999856.1 | NC_009785.1 |
| *Streptococcus intermedius* B196 | WP_021003212.1 | NC_022246.1 |
| *Streptococcus intermedius* C270 | WP_003076142.1 | NC_022237.1 |
| *Streptococcus intermedius* FDAARGOS_233 | WP_082312092.1 | NZ_CP020433.2 |
| ***Streptococcus intermedius* JTH08** | **WP_003076142.1** | **NC_018073.1** |
| *Streptococcus intermedius* NCTC 11324 | WP_003076142.1 | NZ_LS483436.1 |
| *Streptococcus intermedius* TYG1620 | WP_003076142.1 | NZ_AP014880.1 |
| ***Streptococcus pantholopis* TA 26** | **WP_067060972.1** | **NZ_CP014699.1** |
| ***Streptococcus pasteurianus* ATCC 43144** | **WP_003063947.1** | **NC_015600.1** |
| *Streptococcus pasteurianus* NCTC 13784 | WP_111718197.1 | NZ_LS483462.1 |
| *Streptococcus salivarius* ATCC 27945 | WP_084914613.1 | NZ_CP015282.1 |
| *Streptococcus salivarius* HSISS4 | WP_021144275.1 | NZ_CP013216.1 |
| *Streptococcus salivarius* JF | WP_022496455.1 | NZ_CP014144.1 |
| ***Streptococcus salivarius* JIM8777** | **WP_014634402.1** | **NC_017595.1** |
| *Streptococcus salivarius* NCTC 8618 | WP_022496455.1 | NZ_CP009913.1 |
| *Streptococcus* sp. FDAARGOS_192 | WP_080610467.1 | NZ_CP020431.2 |
| ***Streptococcus* sp. HSISS1** | **EQC64892.1** | **CM002132.1** |
| ***Streptococcus* sp. Z15** | **WP_116877779.1** | **NZ_CP031733.1** |
| *Streptococcus thermophilus* APC151 | WP_014608381.1 | NZ_CP019935.1 |
| ***Streptococcus thermophilus* JIM 8232** | **WP_014621674.1** | **NC_017581.1** |
| *Streptococcus thermophilus* LMD-9 | ABJ66323.1 | CP000419.1 |
| *Streptococcus thermophilus* MN-BM-A01 | WP_014727436.1 | NZ_CP012588.1 |
| *Streptococcus thermophilus* MN-ZLW-002 | WP_014727436.1 | NC_017927.1 |
| *Streptococcus thermophilus* MTH17CL396 | ETW89989.1 | CM002371.1 |
| *Streptococcus thermophilus* ND03 | WP_014608381.1 | NC_017563.1 |
| *Streptococcus thermophilus* SMQ-301 | WP_046206469.1 | NZ_CP011217.1 |
| *Streptococcus thermophilus* TH1477 | WP_071417354.1 | NZ_CM003135.1 |
| ***Tumebacillus algifaecis* THMBR28** | **WP_094236481.1** | **NZ_CP022657.1** |
| ***Tumebacillus avium* AR23208** | **WP_087458314.1** | **NZ_CP021434.1** |

* Species listed in bold are represented in Fig. 1.
