# Supplementary material for: Discovery and characterization of a Gram-positive Pel polysaccharide biosynthetic gene cluster
Source: PLoS Pathog. 2020 Apr 1;16(4):e1008281. doi: 10.1371/journal.ppat.1008281 (PMC7112168; doi:10.1371/journal.ppat.1008281)
Supplement: S2 Table — List of E. coli and B. cereus strains generated and used in this study. (DOCX) [file ppat.1008281.s011.docx]

**Table S2: Bacterial strains and plasmids used in this study**

| **Strain** | **Description*** | **Source** | |
| --- | --- | --- | --- |
| ***E. coli*** | | | |
| DH5α | Cloning strain; F^–^ Φ80*lacZ*ΔM15 Δ(*lacZYA*-*argF*) U169 *recA1* *endA1 hsdR17* (r_K_^–^, m_K_^+^) *phoA supE44* λ^–^ *thi*-1 *gyrA96* *relA1* | Invitrogen | |
| EC135 | Strain lacking endogenous restriction modification systems and DNA methyltransferases; TOP10 Δ*dam* Δ*dcm* Δ*hsd* Δ*mcrBC* Δ*mcrA* Δ*mrr* | [1] | |
| BL21CodonPlus^TM^  (DE3)-RP | Protein expression strain; F^-^ *ompT* *hsdS*(rB^−^ mB^−^) *dcm*^+^ Tet^r^ *gal*λ (DE3) *end*AHte *met*A∷Tn5(Kan^r^) [*arg*U *ile*Y *leu*W Cam^r^] | Stratagene | |
| ***B. cereus*** | | | |
| ATCC 10987 | Wild-type strain | A.J. Clarke | |
| ATCC 10987 Δ*pelA_H_* | ATCC 10987 with an unmarked, non-polar deletion of *pelA_H_* (*BCE_5582*) | This study | |
| ATCC 10987 Δ*pelD* | ATCC 10987 with an unmarked, non-polar deletion of *pelD* (*BCE_5583*) | This study | |
| ATCC 10987 Δ*pelE* | ATCC 10987 with an unmarked, non-polar deletion of *pelE* (*BCE_5584*) | This study | |
| ATCC 10987 Δ*pelA_DA_* | ATCC 10987 with an unmarked, non-polar deletion of *pelA_DA_* (*BCE_5585*) | This study | |
| ATCC 10987 Δ*pelF* | ATCC 10987 with an unmarked, non-polar deletion of *pelF* (*BCE_5586*) | This study | |
| ATCC 10987 Δ*pelG* | ATCC 10987 with an unmarked, non-polar deletion of *pelG* (*BCE_5587*) | This study | |
| ATCC 10987 Δ*cdgF* | ATCC 10987 with an unmarked, non-polar deletion of *cdgF* (*BCE_0696*) | This study | |
| ATCC 10987 Δ*cdgE* | ATCC 10987 with an unmarked, non-polar deletion of *cdgE* (*BCE_3781*) | This study | |
| ATCC 10987 *pelD*^R363A^ | ATCC 10987 with a mutation of arginine 363 encoded by *pelD* (*BCE_5583*) to alanine | This study | |
| ATCC 10987 *pelD*^D366A^ | ATCC 10987 with a mutation of aspartic acid 366 encoded by *pelD* (*BCE_5583*) to alanine | This study | |
| ATCC 10987 *pelD*^R395A^ | ATCC 10987 with a mutation of arginine 395 encoded by *pelD* (*BCE_5583*) to alanine | This study | |
| **Plasmid** | **Description** | **Source** | |
| **Recombinant protein expression** | | | |
| pM.Bce | Arabinose inducible vector to express *B. cereus* ATCC 10987 DNA methyltransferases; methylates co-replicating plasmids to overcome the restriction barrier of ATCC 10987; Spc^R^ | | [1] |
| pET-24a(+) | IPTG inducible protein expression vector encoding a C-terminal hexahistidine tag, Kan^R^ | | Novagen |
| pET-28a(+) | IPTG inducible protein expression vector encoding an N-terminal hexahistidine tag and thrombin cleavage site, Kan^R^ | | Novagen |
| pET24a::PelA_H_*^Bc^* | *B. cereus* ATCC 10987 PelA_H_ (BCE_5582), excluding the predicted signal sequence (residues 1-21), inserted between the BamHI and XhoI sites of pET-24a(+) | | This study |
| pET24a::PelA_H_*^Bc-^*^E213A^ | pET24a::PelA_H_*^Bc^* with a mutation of glutamate 213 to alanine | | This study |
| pET28a::PelA_47-303_ | *P. aeruginosa* PAO1 *pelA*, encoding the glycoside hydrolase domain (residues 47-303), inserted between the NdeI and XhoI sites of pET-28a(+) | | [2] |
| pET28a::PelA_47-303_^E218A^ | pET28a::PelA_47-303_ with a mutation of glutamate 218 to alanine | | [2] |
| pET28a::PelD_150-407_ | *B. cereus* ATCC 10987 *pelD* (*BCE_5583*), encoding the predicted cytoplasmic domain of the protein (residues 150-407), inserted between the NheI and XhoI sites of pET-28a(+) | | This study |
| pET28a::PelD_150-407_^R363A^ | pET28a::PelD_150-407_ with a mutation of arginine 363 to alanine | | This study |
| pET28a::PelD_150-407_^D366A^ | pET28a::PelD_150-407_ with a mutation of aspartic acid 366 to alanine | | This study |
| pET28a::PelD_150-407_^R395A^ | pET28a::PelD_150-407_ with a mutation of arginine 395 to alanine | | This study |
| **Allelic exchange** | | | |
| pMAD | *E. coli* – *Bacillus* shuttle vector, encoding the thermostable β-galactosidase *bgaB* from *Bacillus stearothermophilus* driven by the constitutive P*clpB* promoter; Erm^R^, Amp^R^ | | [3] |
| pMAD::Δ*pelA_H_* | *B. cereus* ATCC 10987 Δ*pelA_H_* (*BCE_5582*) cloned between the BamHI and NcoI sites of pMAD | | This study |
| pMAD::Δ*pelD* | *B. cereus* ATCC 10987 Δ*pelD* (Δ*BCE_5583*) cloned between the BamHI and SmaI sites of pMAD | | This study |
| pMAD::Δ*pelE* | *B. cereus* ATCC 10987 Δ*pelE* (Δ*BCE_5584*) cloned between the BamHI and SmaI sites of pMAD | | This study |
| pMAD::Δ*pelA_DA_* | *B. cereus* ATCC 10987 Δ*pelA_DA_* (Δ*BCE_5585*) cloned between the BamHI and SmaI sites of pMAD | | This study |
| pMAD::Δ*pelF* | *B. cereus* ATCC 10987 Δ*pelF* (Δ*BCE_5586*) cloned between the KpnI and BamHI sites of pMAD | | This study |
| pMAD::Δ*pelG* | *B. cereus* ATCC 10987 Δ*pelG* (Δ*BCE_5587*) cloned between the SalI and SmaI sites of pMAD | | This study |
| pMAD::Δ*cdgF* | *B. cereus* ATCC 10987 Δ*cdgF* (Δ*BCE_0696*) cloned between the BamHI and SmaI sites of pMAD | | This study |
| pMAD::Δ*cdgE* | *B. cereus* ATCC 10987 Δ*cdgE* (Δ*BCE_3781*) cloned between the SalI and SmaI sites of pMAD | | This study |
| pMAD::*pelD*^R363A^ | *B. cereus* ATCC 10987 *pelD* (*BCE_5583*), with a mutation of arginine 363 to alanine, cloned between the BamHI and EcoRI sites of pMAD | | This study |
| pMAD::*pelD*^D366A^ | *B. cereus* ATCC 10987 *pelD* (*BCE_5583*), with a mutation of aspartic acid 366 to alanine, cloned between the BamHI and EcoRI sites of pMAD | | This study |
| pMAD::*pelD*^R395A^ | *B. cereus* ATCC 10987 *pelD* (*BCE_5583*), with a mutation of arginine 395 to alanine, cloned between the BamHI and EcoRI sites of pMAD | | This study |
| **Complementation in *B. cereus*** | | | |
| pAD123 | *E. coli* – *Bacillus cereus* shuttle vector, encodes a promoterless copy of GFPmut3a for promoter screening; Cam^R^, Amp^R^ | | [4] |
| pHCMC04 | *E. coli* – *Bacillus subtilis* shuttle vector, contains the xylose-inducible *xylR*-P*_xylA_* promoter cassette; Cam^R^, Amp^R^ | | [5] |
| pAD123-P_xyl_ | pAD123 with the *xylR*-P*_xylA_* cassette from pHCMC04 cloned between the SacI and BamHI sites of pAD123; contains a multiple cloning site (EcoRV-KpnI-NheI-NotI-SmaI-BamHI) immediately downstream of P*_xylA_* | | This study |
| pAD123-P_xyl_::*pelA_H_* | *B. cereus* ATCC 10987 *pelA_H_* (*BCE_5582*) fused to a synthetic RBS (5’-TAAGGAGGAAGCAGGT-3’) cloned between the EcoRV and BamHI sites of pAD123-P_xyl_ | | This study |
| pAD123-P_xyl_::*pelD* | *B. cereus* ATCC 10987 *pelD* (*BCE_5583*) fused to a synthetic RBS (5’-TAAGGAGGAAGCAGGT-3’) cloned between the KpnI and BamHI sites of pAD123-P_xyl_ | | This study |
| pAD123-P_xyl_::*pelE* | *B. cereus* ATCC 10987 *pelE* (*BCE_5584*) fused to a synthetic RBS (5’-TAAGGAGGAAGCAGGT-3’) cloned between the EcoRV and BamHI sites of pAD123-P_xyl_ | | This study |
| pAD123-P_xyl_::*pelA_DA_* | *B. cereus* ATCC 10987 *pelA_DA_* (*BCE_5585*) fused to a synthetic RBS (5’-TAAGGAGGAAGCAGGT-3’) cloned between the KpnI and BamHI sites of pAD123-P_xyl_ | | This study |
| pAD123-P_xyl_::*pelF* | *B. cereus* ATCC 10987 *pelF* (*BCE_5586*) fused to a synthetic RBS (5’-TAAGGAGGAAGCAGGT-3’) cloned between the KpnI and BamHI sites of pAD123-P_xyl_ | | This study |
| pAD123-P_xyl_::*pelG* | *B. cereus* ATCC 10987 *pelG* (*BCE_5587*) fused to a synthetic RBS (5’-TAAGGAGGAAGCAGGT-3’) cloned between the EcoRV and SmaI sites of pAD123-P_xyl_ | | This study |
| pAD123-P_xyl_::*cdgF* | *B. cereus* ATCC 10987 *cdgF* (*BCE_0696*) fused to a synthetic RBS (5’-TAAGGAGGAAGCAGGT-3’) cloned between the KpnI and BamHI sites of pAD123-P_xyl_ | | This study |
| pAD123-P_xyl_::*cdgE* | *B. cereus* ATCC 10987 *cdgE* (*BCE_3781*) fused to a synthetic RBS (5’-TAAGGAGGAAGCAGGT-3’) cloned between the EcoRV and BamHI sites of pAD123-P_xyl_ | | This study |

*Amp, ampicillin; Cam, chloramphenicol; Kan, kanamycin; Erm, erythromycin; Spc, spectinomycin

**References**

1. Zhang G, Wang W, Deng A, Sun Z, Zhang Y, Liang Y, et al. A mimicking-of-DNA-methylation-patterns pipeline for overcoming the restriction barrier of bacteria. PLoS Genet. 2012;8: e1002987. doi:10.1371/journal.pgen.1002987

2. Baker P, Hill PJ, Snarr BD, Alnabelseya N, Pestrak MJ, Lee MJ, et al. Exopolysaccharide biosynthetic glycoside hydrolases can be utilized to disrupt and prevent Pseudomonas aeruginosa biofilms. Sci Adv. 2016;2: e1501632. doi:10.1126/sciadv.1501632

3. Arnaud M, Chastanet A, Débarbouillé M. New vector for efficient allelic replacement in naturally nontransformable, low-GC-content, gram-positive bacteria. Appl Environ Microbiol. 2004;70: 6887–6891. doi:10.1128/AEM.70.11.6887-6891.2004

4. Dunn AK, Handelsman J. A vector for promoter trapping in Bacillus cereus. Gene. 1999;226: 297–305. doi:10.1016/s0378-1119(98)00544-7

5. Nguyen HD, Nguyen QA, Ferreira RC, Ferreira LCS, Tran LT, Schumann W. Construction of plasmid-based expression vectors for Bacillus subtilis exhibiting full structural stability. Plasmid. 2005;54: 241–248. doi:10.1016/j.plasmid.2005.05.001
