# Supplementary material for: Discovery and characterization of a Gram-positive Pel polysaccharide biosynthetic gene cluster
Source: PLoS Pathog. 2020 Apr 1;16(4):e1008281. doi: 10.1371/journal.ppat.1008281 (PMC7112168; doi:10.1371/journal.ppat.1008281)
Supplement: S3 Table — List of primers used for sequencing, allelic exchange, generation of complementation vectors and recombinant protein expression. (DOCX) [file ppat.1008281.s012.docx]

**Table S3: Primers used in this study**

| **Primers** | **Sequence*** |
| --- | --- |
| **Vectors for recombinant protein production** | |
| PelA_H_*^Bc^* pET-F | GGG **GGA TCC** AAT GTC GTG GAA CCA GTT TTA AAA |
| PelA_H_*^Bc^* pET-R | GGG **CTC GAG** TTT ATC ATA AAT ATC ATT CGG ATT AAT TG |
| PelA_H_*^Bc^* E213A upR | T ATT GAA GTC CgC CCA CAA TAC A |
| PelA_H_*^Bc^* E213A downF | T GTA TTG TGG GcG GAC TTC AAT A |
| PelD_Bc_ 150F | CG **GCT AGC** GAA TCA AAT AAT CAC TTA TCT AAA ATG TAT C |
| PelD_Bc_ 407R | GC **CTC GAG** TTA GCA TAC CAC TCC TTT AGA AGA AA |
| PelD_Bc_ R363A F | AAA GAT CAT TTG gcT GAT ATC GAT ATA |
| PelD_Bc_ R363A R | TAT ATC GAT ATC Agc CAA ATG ATC TTT |
| PelD_Bc_ D366A F | TTG CGT GAT ATC GcT ATA TTC GGT TA |
| PelD_Bc_ D366A R | TA ACC GAA TAT AgC GAT ATC ACG CAA |
| PelD_Bc_ R395A F | A GTC CAA ACT gcT ATA CAA AAC GC |
| PelD_Bc_ R395A R | GC GTT TTG TAT Agc AGT TTG GAC T |
| **Allelic exchange vectors** |  |
| ΔpelA_H_ upF | GTG **GGA TCC** GTA TTC GAT CTG TTT TTC GTA CCG |
| ΔpelA_H_ upR | *ATA AAT ATC ATT CGG ATT AAT TGT AGG* AAA GTA CAT GGA ACG TTT CCA CTC |
| ΔpelA_H_ downF | CCT ACA ATT AAT CCG AAT GAT ATT TAT |
| ΔpelA_H_ downR | GGA **CCA TGG** CAA CCC TCG GAA CGG GAA TC |
| ΔpelD upF | GGT **GGA TCC** CTA AAT AGG TCA GAC TTC CTA CC |
| ΔpelD upR | *TTA GCA TAC CAC TCC TTT AGA* TAA ATT AGA TTG TCT ATA GTG CAT |
| ΔpelD downF | TCT AAA GGA GTG GTA TGC TAA |
| ΔpelD downR | GAG **CCC GGG** AGT TGT ATT CTC ATC TAG CGT AAT AT |
| ΔpelE upF | GGG **GGA TCC** GAT GAT ATAT TAC TGT ACT TTT ATT CAT TAG |
| ΔpelE upR | *AAT GTG TGC GCC TCC AAA CTG* AAA GTA AAG AAG GAG TAA AAT CAT |
| ΔpelE downF | CAG TTT GGA GGC GCA CAC ATT |
| ΔpelE downR | GTG **CCC GGG** TTC TCT CCC AAA TAA TAA ATA GGT ATT |
| ΔpelA_DA_ upF | GAG **GGA TCC** ACT GGT AAA AAT ACC TTA CCT CAA TC |
| ΔpelA_DA_ upR | *TAC CCC TTT AAT TGG AAT TGT AGC* GAT ATA TAT ATT AAT CCC CTT TTT CAA |
| ΔpelA_DA_ downF | GCT ACA ATT CCA ATT AAA GGG GTA |
| ΔpelA_DA_ downR | GTG **CCC GGG** CTT TTA CCC TTG AAG AAT AGT AGT CT |
| ΔpelF upF | TAG TAC AGA **GTC GAC** GGA AGT AAA AGT AAC GGC AGT TTA AT |
| ΔpelF upR | *ATA CCT GCC ATG TTT TCG CAC C*GC CAC TGA CAA AAG GAT AAC TTC C |
| ΔpelF downF | GGT GCG AAA ACA TGG CAG GTA T |
| ΔpelF downR | TAG TAC AGA **CCC GGG** GAT CCG ATT AAA AAT GCC AGT GCA |
| ΔpelG upF | ACG **GTC GAC** GCC AAA ACA TAA TTC AGA TAA AC |
| ΔpelG upR | *TTA TTT CCC GTT AAG TTT ATT AGC* TCG AAA TCC TAT ACC TGC CAT |
| ΔpelG downF | GCT AAT AAA CTT AAC GGG AAA TAA |
| ΔpelG downR | TAT **CCC GGG** CAG ATA GAG AAT GGA GTA TCG |
| ΔcdgF upF | AGG **GGA TCC** TTC TTC TGA ACG ATG GAG ATGC |
| ΔcdgF upR | *TAA CTT TTC TTT TAA CTC TCC TAC AGA* TGC ATG ACC TCT TTG TTC TAG CAT |
| ΔcdgF downF | TCT GTA GGA GAG TTA AAA GAA AAG TTA |
| ΔcdgF downR | GGT **CCC GGG** GTT TTA ACA GCT TTT GTT ATC GGG |
| ΔcdgE upF | GGG **GTC GAC** TAT TTC TGT TAC TCT ATG AGC GTC T |
| ΔcdgE upR | *GAG AAG AGT AGA AAA TGA ATC AGC* TGT CGC AAT TAA TAT ACA TAG TTG TAA |
| ΔcdgE downF | GCT GAT TCA TTT TCT ACT CTT CTC |
| ΔcdgE downR | GAG **CCC GGG** TGA AGG GAT CGT GGA TAT AAG C |
| pelD I-site F | GGC **GGA TCC** CGA AAG AAG GGA TTT TTT ATG CAC TA |
| pelD I-site R | GCC **GAA TTC** CTT GTT GCC CTA ATG TAT TAT CCG |
| pelD R363A upR | A TAT ATC GAT ATC Agc CAA ATG ATC TTT TAA AAT AGA ATT TAT CAT TTC TAA AGA A |
| pelD R363A downF | A GAT CAT TTG gcT GAT ATC GAT ATA TTC GGT TAT AGC ACG ACT AAA CAA |
| pelD D366A upR | ATA TAG CgA TAT CAC GCA AAT GAT CTT TTA AAA TAG AAT TTA TCA TTT CTA AAG AA |
| pelD D366A downF | A GAT CAT TTG CGT GAT ATC GcT ATA TTC GGT TAT AGC ACG ACT AAA CAA |
| pelD R395A upR | C GTT TTG TAT Agc AGT TTG GAC TGG TAA TAA AAA CTT TTC TTC AGT GCC A |
| pelD R395A downF | CA GTC CAA ACT gcT ATA CAA AAC GCT CTT TCT TCT AAA GGA GTG GTA |
| **Complementation vectors** | |
| xylR-PxylA F | GGG **GAG CTC** CTA ACT TAT AGG GGT AAC ACT TA |
| xylR-PxylA R | GAA **GGA TCC** CAT TTC CCC CTT TGA TTT TTA GA |
| Pxyl MCS F | AG CGC GGC CGC CCC GGG GGA TCC TCT AGA TTT AAG AAG GAG |
| Pxyl MCS R | AGC GGT ACC GAT ATC CAT TTC CCC CTT TGA TTT AAG TGA A |
| pelA_H_ F | GGT **GAT ATC *TAA GGA GGA AGC AGG T***AT GGA GTG GAA ACG TTC CAT GTA |
| pelA_H_ R | GGT **GGA TCC** CTA TTT ATC ATA AAT ATC ATT CGG ATT AA |
| pelD F | GAG **GGT ACC *TAA GGA GGA AGC AGG T***AT GCA CTA TAG ACA ATC TAA TTT ATT AC |
| pelD R | GAG **GGA TCC** TTA GCA TAC CAC TCC TTT AGA AGA |
| pelE F | GGG **GAT ATC *TAA GGA GGA AGC AGG T***AT GAT TTT ACT CCT TCT TTA CTT TAT T |
| pelE R | GTG **GGA TCC** TTA ATC CCC TTT TTC AAT GTG TGC |
| pelA_DA_ F | GAG **GGT ACC *TAA GGA GGA AGC AGG T***TT GAA AAA GGG GAT TAA TAT ATA TAT CG |
| pelA_DA_ R | GTG **GGA TCC** CTA TAC CCC TTT AAT TGG AAT TGT AG |
| pelF F | GAG **GGT ACC *TAA GGA GGA AGC AGG T***AT GAG AAT AGG TTT AGT CGT TGA AG |
| pelF R | GGT **GGA TCC** CTA TAC CTG CCA TGT TTT CGC A |
| pelG F | GGG **GAT ATC *TAA GGA GGA AGC AGG T***AT GGC AGG TAT AGG ATT TCG ATT A |
| pelG R | GAG **CCC GGG** TTA TTT CCC GTT AAG TTT ATT AGC TAA |
| cdgF F | GAG **GGT ACC *TAA GGA GGA AGC AGG T***AT GCT AGA ACA AAG AGG TCA TGC A |
| cdgF R | GTG **GGA TCC** TTA TAT GAA ATC TGT AGT TAA CTT TTC |
| cdgE F | GGT **GAT ATC *TAA GGA GGA AGC AGG T***GT GAT TTT ATT GAA ATT AAA TAA AAA T |
| cdgE R | GGT **GGA TCC** CTA CAG AGA ATG AAC ACC TTT T |
| **Sequencing primers** | |
| T7 | TAA TAC GAC TCA CTA TAG GG |
| T7ter | GCT AGT TAT TGC TCA GCG G |
| pMAD SEQ-F | GCA ACG CGG GCA TCC CGA TG |
| pMAD SEQ-R | CCC AAT ATA ATC ATT TAT CAA CTC TTT TAC ACT TAA ATT TCC |
| pAD123-P_xyl_ SEQ-F | GAT AGT TGA TGG ATA AAC TTG TTC |
| pAD123-P_xyl_ SEQ-R | CAA GAA TTG GGA CAA CTC CAG |
| pelA_DA_ SEQ-int | GTT CAA TCA CTG GGT ACT ATT TTC |
| cdgF SEQ-int | ATG CTT TCA GGC ACC ATT TAT GC |
| cdgE SEQ-int-1 | CTG AAA CGA TAA CAT TAT TAC GTC |
| cdgE SEQ-int-2 | GAA AAT TGG GAA CCG TGC CTT T |
| cdgE SEQ-int-3 | CAA GTT GAT AGC GAA TCT AAC GAC |

*Restriction sites are bolded; regions of complementary to the target amplicon are underlined; regions of reverse complementarity (to facilitate splicing) are italicized; lowercase letters denote a nucleotide substitution; synthetic ribosomal binding sites are in bold italics.
